# Discussing patient's lifestyle choices in the consulting room: analysis of GP-patient consultations between 1975 and 2008

**DOI:** 10.1186/1471-2296-11-87

**Published:** 2010-11-09

**Authors:** Janneke Noordman, Peter Verhaak, Sandra van Dulmen

**Affiliations:** 1 Netherlands Institute for Health Services Research (NIVEL), PO Box 1568, 3500 BN Utrecht, The Netherlands

## Abstract

**Background:**

The increasing prevalence of chronic diseases and the growing understanding that lifestyle behaviour plays an essential role in improving overall health suggest a need for increased attention to lifestyle choices in the consulting room.

This study aims to examine whether or not healthy and unhealthy lifestyle choices of patients are currently being discussed more often in primary care consultations than in former decades. Furthermore, we are interested in GPs' approach to lifestyle behaviour during consultations. Lastly, we examine whether lifestyle behaviour is discussed more with certain patients during consultations, depending on gender, age and educational background.

**Method:**

We analysed video-recordings of medical consultations, collected between 1975 and 2008 in Dutch GP practices. Data were analysed using logistic regression.

**Results:**

This study shows that discussion of smoking behaviour and physical activity has increased somewhat over time. A change in discussion of nutrition and alcohol is, however, less clear. Overall, alcohol use is the least discussed and physical activity the most discussed during consultations. GPs mainly refer to lifestyle when it is relevant to the patient's complaints (symptom approach). GPs' approach to lifestyle behaviour did not change over time. In general, lifestyle behaviour is discussed more with older, male patients (except for nutrition). GPs talk about lifestyle behaviour with patients from different educational backgrounds equally (except for physical activity).

**Conclusion:**

In recent years there is greater awareness of a healthy lifestyle, which is reflected to a limited extent in this study. Still, lifestyle behaviour is discussed in only a minority of consultations. GPs do not refer to lifestyle behaviour as a routine procedure, i.e. do not include it in primary prevention. This highlights the importance of the introduction of prevention consultations, where GPs can discuss lifestyle issues with patients who do not (yet) have risk symptoms.

## Background

Smoking, poor nutrition, alcohol abuse and physical inactivity are related to chronic diseases like heart and vascular disease, diabetes type II, Chronic Obstructive Pulmonary Disease (COPD), certain cancers and hypertension [1,2]. Changes in lifestyle, such as increased exercise, improved diet, lower alcohol consumption and non smoking can therefore improve overall health [3] and subjective well-being [4]. Governments and health service providers in many countries in the Western world recognize that advice on lifestyle risk factors is essential in the prevention of (chronic) diseases and the improvement of public health [5]. Especially in recent years there is greater awareness of improving lifestyle behaviour [6,7]. For example, in the Netherlands the government has developed a prevention bill, aimed at reducing the incidence of smoking, alcohol abuse, obesity, diabetes (type II) and depression [8,9].

General practitioners (GPs) play an important role in discussing lifestyle factors with their patients. Yet, previous research indicates room for improvement in both the frequency and quality of lifestyle advice given [10,11]. Common barriers for GPs to give advice about lifestyle are lack of confidence in its efficacy as well as a lack of time and financial incentives [12,13].

GPs tend to provide lifestyle advice mainly to patients who are at high risk or already have symptoms of certain diseases. A population approach, discussing lifestyle behaviour as a routine procedure, seems less common according to Swedish and UK research [14,15]. However, it is possible that these research findings do not apply to the situation in Dutch general practice, due to differences in health care systems and in policy on lifestyle behaviour. Furthermore, giving lifestyle advice to the patient is not within GP's task perception; GPs found it less relevant and appropriate than illness management [16]. Recent developments, such as the expected introduction of a prevention consultation and the use of practice nurses in primary care may generate a more pivotal and responsible position for the GP (and practice nurses) regarding patients' lifestyle behaviour [17]. The increasing prevalence of chronic diseases and the growing understanding that lifestyle behaviour plays an essential role in improving overall health [7,9] suggest a need for increased attention to lifestyle choices in the consulting room.

Unhealthy lifestyle behaviour clusters in certain groups. It has a higher prevalence in lower socio-economic groups [11,18,19], and there are indications that it is age and gender-dependent. A previous study showed that male patients from the age of 50 had a healthier lifestyle and their behaviour changes were of more significance than male patients aged between 30 and 49 [20]. Another study found a higher prevalence of alcohol use, smoking and lower physical activity among male patients [21]. It is not clear whether GPs adapt the discussion of lifestyle behaviour to specific patient groups.

To explore whether or not healthy and unhealthy lifestyle is being discussed more often in recent primary care consultations, we analysed consultations between GPs and patients in the Netherlands recorded on video between 1975 and 2008.

In addition, we are interested in the kind of approach (population, high risk or symptom approach) taken by GPs in relation to lifestyle behaviour, whether a GP's approach to lifestyle behaviour changes over time and whether GPs adapt the discussion of lifestyle behaviour to specific patient groups. Three approaches were defined, based on a combination of literature findings [14,15] and our insights: 1. 'Population approach', GPs discuss lifestyle behaviour with all patients; 2. 'High risk approach', discussing lifestyle only with patients with (risk of) chronic diseases; and 3. 'Symptom approach', discussing lifestyle behaviour when it is relevant to the patient's presented symptom, without the patient being at high risk or having a chronic disease (for example asking about smoking habits if the patient is coughing).

To sum up, our research questions are:

1. How often is healthy and unhealthy behaviour of the patient (smoking, nutrition, alcohol consumption, and physical activity) discussed in GP consultations?

2. Has the frequency of discussing lifestyle during GP consultations changed over time?

3. Who takes the initiative (GP or patient) to discuss the patient's lifestyle behaviour? Has the initiative to discuss lifestyle behaviour changed over time?

4. What symptoms do patients show when lifestyle behaviour is discussed and to what extent do GPs use a 'population approach', 'high risk approach' or 'symptom approach' to discuss lifestyle behaviour? Has GPs' approach to discussing lifestyle behaviour changed over time?

5. Is lifestyle behaviour discussed more (or less) with certain patients during primary care consultations; depending on educational background, age group and/or gender?

## Method

We used real-life videotaped GP-patient consultations to observe if and how often (un)healthy behaviour is discussed during consultations. Neither patients nor GPs were aware of the fact that the analysis would focus on communication about lifestyle behaviour. Video recording is an optimal method to observe GP-patient communication; the influence of the video recorder on the participants' behaviour is marginal [22].

Video-recordings were collected as part of eight different studies conducted by NIVEL (Netherlands Institute for Health Services Research): (1) in 1975 [23], (2) in 1977-1979 [24], (3) 1978-1980 [25], (4) 1982-1984 [26], (5) 1989 [27], (6) 1995-1996 [28], (7) 2000-2001 [29] and (8) 2007-2008 [30]. Table 1 describes the characteristics of patients and GPs who participated in the studies from 1975 until 2008. Over the years more female GPs participated in the studies. Other differences between the studies concern the number of participating patients and GPs and the representativeness of the samples. Some studies reflect Dutch GPs regarding practice form (solo, duo, group practice or health centre), age [29,30], sex [29], urbanicity and region [27,29], while other studies represent a local [23,24] or random [25,26,28] sample of Dutch GPs. GP's response ranged between 21% in 1996 and 73% in 2001. Patients' response for the different studies ranged between 77% in 1989 and 88% in 2001.

**Table 1:** Characteristics of GPs and patients in the observed consultations 1975-2008

	1975	1977-1979	1978-1980	1982-1984	1989	1995-1996	2000-2001	2007-2008
Patients	n = 214	n = 345	n = 363	n = 1699	n = 250	n = 442	n = 2082	n = 808
Age mean (SD)	41 (15)	39 (15)	44 (18)	40 (17)	36 (20)	41 (22)	43 (22)	43 (23)
Gender (%)	30% m 70% f	30% m 70% f	42% m 58% f	38% m 62% f	39% m 61% f	38% m 62% f	40% m 60% f	41% m 59% f
GPs	n = 10	n = 9	n = 10	n = 30	n = 17	n = 32	n = 155	n = 40
Gender (%)	100% m	100% m	100% m	97% m 3% f	76% m 24% f	48% m 52% f	77% m 23% f	65% m 35% f

The studies were carried out according to Dutch privacy legislation. The privacy regulation was approved by the Dutch Data Protection Authority. According to Dutch legislation, approval by a medical ethics committee was not required for these observational studies.

### Data collection

In all studies, an unmanned camera was installed for one or two random days in the consulting room of the GP concerned. Consecutive patients who had an appointment with the GP were approached by a researcher in the waiting room, who requested (written) informed consent and handed out the questionnaires. These questionnaires contained information about patients' characteristics (age, gender and educational background), their health, and the importance and performance scores they attribute to the communication with the GP. For some periods these questionnaires also contained additional questions: more detailed questions about health and use of care, opinions about referring and prescribing medication, preferences for care, social support, life events [27,29] preferences for their role in decision making, recall of information and medication adherence [30].

Educational background of the patient is used as a proxy for social economic status (SES).

### Observations

The videotaped consultations were reviewed by two to six observers per study using an observation checklist, which was fairly similar for each time period. For each consultation the observers described whether the GP discussed (un)healthy behaviour of the patient in relation to smoking, alcohol use, nutrition and physical activity (Yes/No). For the 2001 and 2008 studies we additionally took account of whose initiative (GP or patient) it was to discuss lifestyle. See Table 2 for transcribed video fragments showing whether the GP or patient takes the initiative to discuss lifestyle behaviour.

**Table 2:** Video fragments in which the GP or patient takes the initiative to discuss lifestyle behaviour of the patient

1	GP: How are you doing with the smoking? (*Initiative GP*)
	Pt: I'm not!.. smoking anymore.
	GP: Very good!
2	GP: So you are here for the blood pressure check. I see it's been a while.
	Pt: Yes it has. I'm working out now a lot... a lot! (*Initiative patient*)
	GP: You say a lot. Did you lose some weight also?
	Pt: Yes, 33 pounds in total!
	GP: How much do you weigh now?
	Pt: uhmm about 200 pounds.
	GP: Ok. It's quite a lot...losing 33 pounds I mean.
	Pt: Yes, with help from a dietician
	GP: It doesn't matter how you do it! And what do you want to weight? What is your goal?
	Pt: I think 176 pounds would be nice. But now it is difficult.
	GP: Yes, the first pounds are easy, but well every pound counts. You should keep up the good work!

3	Pt: My throat hurts from coughing.
	GP: And you don't touch the cigarettes? (*Initiative GP*)
	Pt: No, because I have a fake cigarette now.
	GP: How is that working for you?
	Pt: I'll get used to it, although it is so heavy
	GP: But you do use it?
	Pt: Yes
	GP: And no more cigarettes?
	Pt: Well, I'm finishing the last ones.
	GP: Ok. But it helps you when you smoke the fake cigarette?
	Pt: Yes, it's just like a normal cigarette, with smoke and everything.

GP = General practitioner, Pt = patient

Furthermore, we registered the symptoms presented during the consultation. Symptoms were described according to the International Classification of Primary Care (ICPC). Since we are interested in the association between the patient's symptoms and the discussion of lifestyle behaviour with GPs, we selected consultations where the patient exhibited only one symptom. This was because when patients exhibited more than one symptom we could not directly relate these to the discussion of lifestyle behaviour. We used the patient's symptoms to identify GP's approach (population, high risk or symptom approach) to lifestyle behaviour.

### Interrater reliability

Observers were trained to observe the behaviours of GPs and patients during consultations in each time period. To compute reliability, 40 of the same consultations were observed by two observers. We calculated the interrater reliability between the observers with Cohen's kappa [31]. The interrater reliability is calculated for the 2007-2008 study. For the other periods (1975-2001) we could not calculate the interrator reliability for the categories of lifestyle behaviour since the different observers coded all different consultations regarding 'lifestyle behaviour'.

### Statistical analyses

We compared smoking, alcohol use, nutrition and physical activity (as dichotomy variables) respectively during the eight periods (time series 1-8), using logistic regression. The time series were used as continuous variables, while correcting for the different periods between the time series. Next, we used 'time series' also as dummy variables (with time serie 1 as reference group) in logistic regression, to give more insight in the different periods when smoking, alcohol use, nutrition and physical activity are discussed (compared to the reference group). Differences in initiatives were analysed using T-test. Differences in patients' educational background, gender and age were analysed using logistic regression (with no formal education and male as reference groups; age was used as continues variable).

We performed analyses using Stata version 10 [32].

## Results

### Interrater reliability

For all the categories of lifestyle behaviour kappa is sufficiently reliable [31] (see Table 3).

**Table 3:** Interrater reliability 2007-2008, Cohen's kappa

	Kappa
**Discussing lifestyle behaviour**	

Smoking	0.79

Alcohol use	0.66

Nutrition	0.73

Physical activity	0.74

### Discussing healthy and unhealthy lifestyle with the patient

Table 4 describes the percentage of consultations in which the GP discusses (un)healthy lifestyle behaviour with the patient and if the discussing of lifestyle changed over time.

**Table 4:** Percentage of consultations in which the GP discusses lifestyle choices with the patient, 1975-2008

Time series/ Behaviour:	1975 (1) (n = 214)	1977-1979 (2) (n = 345)	1978-1980 (3) (n = 363)	1982-1984 (4) (n = 1699)	1989 (5) (n = 250)	1995-1996 (6) (n = 442)	2000-2001 (7) (n = 2082)	2007-2008 (8) (n = 808)	Overall
Smoking*	4.7% CI: 1.8-7.5	2.3% CI: 0.7-3.9	4.1% CI: 2.1-6.2	4.1% CI: 3.2-5.1	10.4% CI: 6.6-14.2	7% CI: 4.6-9.4	8.3% CI: 7.1-9.5	8.3% CI: 6.3-10.2	6.2%

Alcohol use*	1.4% CI: 0.0-2.9	1.4% CI: 0.2-2.7	1.9% CI: 0.5-3.3	2.0% CI: 1.3-2.7	3.2% CI: 1.0-5.4	4.3% CI: 2.4-6.2	2.7% CI: 2.0-3.4	3.5% CI: 2.2-4.7	2.6%

Nutrition*	8.8% CI: 5.0-12.7	7.5% CI: 4.7-10.3	10.5% CI: 7.3-13.6	8.1% CI: 6.8-9.4	10% CI: 6.3-13.7	13.1% CI: 10-16.3	13.3% CI: 11.8-14.7	11.1% CI: 9.0-13.3	10.3%

Physical* activity	5.6% CI: 2.5-8.7	5.2% CI: 2.9-7.6	6.3% CI: 3.8-8.9	8.4% CI: 7.0-9.7	16.4% CI: 11.8-21.0	13.1% CI: 10-16.3	27.2% CI: 25.3-29.1	23% CI: 20.1-26.0	13.2%

* Significant time trend: discussing of lifestyle behaviour increases over time, logistic regression (time series as continues variable), Smoking = OR: 1.03 & CI: 1.02-1.04; Alcohol use = OR: 1.02 & CI: 1.01-1.04; Nutrition = OR: 1.02 & CI:1.01-1.03; Physical activity = OR: 1.06 & CI: 1.06-1.07

OR = Odds Ratio, CI = 95% Confidence Interval

Table 5 describes also the discussing of lifestyle over time, but with 1975 as reference year.

**Table 5:** Consultations in which the GP discusses lifestyle choices with the patient, 1975-2008, logistic regression (Odds Ratio & 95% Confidence Interval)

Time series/Behaviour:	1975 (1) (n = 214)	1977-1979 (2) (n = 345)	1978-1980 (3) (n = 363)	1982-1984 (4) (n = 1699)	1989 (5) (n = 250)	1995-1996 (6) (n = 442)	2000-2001 (7) (n = 2082)	2007-2008 (8) (n = 808)
Smoking	Ref	OR: 0.5 CI: 0.2-1.2	OR: 0.9 CI: 0.4-2.0	OR: 0.9 CI: 0.4-1.7	OR: 2.4 CI: 1.1-5.1*	OR: 1.5 CI: 0.7-3.2	OR: 1.8 CI: 1.0-3.6	OR:1.8 CI: 0.9-3.6

Alcohol use	Ref	OR:1.03 CI: 0.2-4.4	OR:1.4 CI: 0.4-5.4	OR:1.4 CI: 0.4-4.7	OR: 2.3 CI: 0.6-8.9	OR:3.1 CI: 0.9-10.8	OR: 2.0 CI: 0.6-6.4	OR: 2.5 CI: 0.8-8.4

Nutrition	Ref	OR: 0.8 CI: 0.5-1.6	OR: 1.2 CI: 0.7-2.2	OR: 0.9 CI: 0.5-1.5	OR: 1.1 CI: 0.6-2.1	OR:1.6 CI: 0.9-2.7	OR: 1.6 CI: 1.0-2.6	OR: 1.3 CI: 0.8-2.2

Physical activity	Ref	OR: 09 CI: 0.4-2.0	OR: 1.1 CI: 0.6-2.3	OR: 1.5 CI: 0.8-2.8	OR: 3.3 CI: 1.7-6.5*	OR: 2.5 CI: 1.3-4.8*	OR: 6.3 CI: 3.5-11.4*	OR: 5.0 CI: 2.7-9.2*

Ref = reference group, OR = Odds Ratio, CI = Confidence Interval

* Significant difference (P < 0.05) compared to reference group (time serie 1), logistic regression (time series as dummy variables)

GPs discussed 'smoking behaviour' with the patient significantly more often during consultations in more recent years (time series 5-8) compared with previous years (time series 1-4). Smoking behaviour is discussed in 6.2 percent of the consultations on average.

It appears that the odds that GPs discuss smoking behaviour increase by a factor of 1.03 over time (95% CI 1.02-1.04). This means that for each additional year, there is a 3% increase in the odds of discussing smoking behaviour (Table 4). Table 5 shows that smoking behaviour is only significantly more often discussed during 1989 (time serie 5) compared to 1975 (time serie 1).

Alcohol use is discussed in 2.6 percent of the consultations. The odds that GPs discuss alcohol use increase by a factor of 1.02 over time (95% CI 1.01-1.04) (see Table 4). In contrast, Table 5 describes no significant difference over the years in discussing alcohol use (compared to 1975).

During 10.3 percent of GP-patient consultations nutrition is discussed. For each additional year there is a 2% increase in the likelihood of discussing nutrition (95% CI 1.01-1.03) (Table 4). Table 5 shows no significant difference over the years in discussing nutrition (compared to 1975).

Physical activity is discussed in 13.2 percent of consultations. The probability that GPs discuss physical activity increases by a factor of 1.06 over time (95% CI 1.06-1.07) (see Table 4). Table 5 describes that physical activity is significantly more discussed from 1989 until 2008 (time series 5-8) compared to 1975 (time serie 1).

### Initiative to discuss lifestyle

Table 6 describes who takes the initiative (GP or patient) to discuss lifestyle behaviour, during consultations in 2000-2001 and 2007-2008.

**Table 6:** Initiative to discuss lifestyle behaviour (consultation level)

Behaviour:	Discussed?	2000-2001 (n = 2082)	2007-2008 (n = 808)
	Not discussed	91.7%	91.7%
	
Smoking	GP's Initiative	6.0%*	6.5%*
	
	Patient's Initiative	2.3%	1.8%

	Not discussed	97.3%	96.5%
	
Alcohol use	GP's Initiative	1.4%	2.1%*
	
	Patient's Initiative	1.3%	1.4%

	Not discussed	86.7%	88.9%
	
Nutrition	GP's Initiative	6.0%	5.4%
	
	Patient's Initiative	7.3%	5.7%

	Not discussed	72.8%	77%
	
Physical activity	GP's Initiative	9.8%*	9.4%
	
	Patient's Initiative	17.4%	13.6%

*Significant difference between GP's initiative and Patient's initiative, T-test, P(<0.05).

Only in a small proportion of the consultations GPs or patients take the initiative to discuss lifestyle behaviour. When lifestyle behaviour is discussed, GPs mostly take the initiative to discuss smoking behaviour and alcohol use. The initiative to discuss physical activity is more often taken by the patients themselves. There are no significant changes over time regarding the initiative to discuss lifestyle behaviour.

### Symptoms and approach to discussing lifestyle behaviour

Table 7 shows the symptoms for which lifestyle items (smoking, alcohol use, nutrition and physical activity) are discussed during a GP-patient consultation and GP's approach to patient's lifestyle behaviour. There were no significant differences in the kind of symptom (ICPC chapter) from 1975 until 2008 when discussing lifestyle items; therefore we make no distinction between the years.

**Table 7:** Patient's symptom (ICPC) when the GP discusses smoking, alcohol use, nutrition and physical activity during a consultation, 1975-2008

Patient's symptom (ICPC-chapter)*	Smoking (n = 172)	Alcohol use (n = 62)	Nutrition (n = 280)	Physical activity (n = 491)
General	**24 **(14.0%)	**12 **(19.4%)	**26 **(9.3%)	**38 **(7.7%)

Blood	3	**10 **(16.1%)	4	3

Digestive	11	-	**91 **(32.5%)	16

Eye	2	-	-	3

Ear	-	-	5	13

Circulatory	**19 **(11%)	8	**29 **(10.4%)	22

Musculoskeletal	17	6	20	**281 **(57.2%)

Neurological	3	3	9	17

Psychological	9	**11 **(17.7%)	11	11

Respiratory	**58 **(33.7%)	2	22	**33 **(6.7%)

Skin	3	1	18	26

Metabolic, endocrine, nutrition	12	5	21	10

Urological	1	-	1	1

Pregnancy, family planning	6	4	8	4

Female genital	2	-	10	8

Male genital	1	-	2	1

Social problems	1	-	2	4

				

GP's approach to patient's lifestyle behaviour:	Smoking (n = 172)	Alcohol use (n = 62)	Nutrition (n = 280)	Physical activity (n = 491)

Symptom approach	134	45	251	407

High risk approach	38	17	29	84

Population approach**	-	-	-	-

*Consultations where the patients' show only one symptom (ICPC).

** A population approach is not visible since GPs discussed lifestyle behaviour only in a minority of the consultations.

Most of the patients had presented with respiratory complaints (in particular throat and breathing problems) when GPs provided advice on smoking behaviour, followed by 'general' complaints (mainly fatigue and medication issues) and circulatory complaints (especially related to heart and vascular diseases and heart medication).

If GPs discuss alcohol use, patients tend to exhibit 'general' symptoms (in particular fatigue and medication issues), blood related symptoms (especially enlarged lymph node) and psychological symptoms (mainly drug misuse, stress and anxiety).

When GPs provide advice about nutrition to patients during a consultation, patients mainly have digestive complaints (in particular abdominal pains, stomach ache and diarrhoea), followed by circulatory complaints (especially hypertension and discussing heart research) and general complaints (mainly fatigue and fever).

Most patients present with musculoskeletal complaints (especially back, knee and shoulder symptoms) when the GP discusses physical activity during a consultation. This is followed by general complaints (mainly fatigue and medication issues) and respiratory complaints (especially breathing problems and hyperventilation).

GPs in this study are shown to mainly use a 'symptom' approach to a patient's lifestyle behaviour. They discuss lifestyle behaviour when relevant to the patient's complaint; for example discussing nutrition when the patient has a stomach ache and smoking cessation when the patient has breathing problems. Lifestyle behaviour is also, to a small extent, discussed with patients at risk of or suffering from a chronic disease. An example of the 'high risk' approach in this study was the discussion of physical activity and nutrition with overweight patients and smoking behaviour with patients who have heart problems. The 'population approach' is not visible in this study, since lifestyle behaviour was only discussed in a minority of the consultations.

### Educational background, age and gender of patients while lifestyle behaviour is discussed

Tables 8 and 9 show the educational background, age and gender of patients with whom lifestyle behaviour is discussed.

**Table 8:** Patients' educational background when lifestyle behaviour is discussed, 2000-2008

Discussing/Educational background:	Smoking (n = 189)	Alcohol (n = 65)	Nutrition (n = 270)	Physical activity (n = 579)	Total
No formal education (ref)	14 (6%)	2 (0.9%)	29 (12.6%)	33 (14.3%)	231

Primary school	34 (7.1%)	8 (1.7%)	50 (10.4%)	102 (21.3%)	479

High school or vocational education	101 (7.4%)	38 (2.8%)	138 (10.2%)	301 (22.2%)	1358

College or university	33 (7.6%)	16 (3.7%)	42 (9.7%)	115 (26.6%)*	432

*Significant difference, logistic regression ('no formal education' as reference group), OR: 1.9 & CI: 1.2-3.1.

**Table 9:** Patients' age and gender when lifestyle behaviour is discussed, 1975-2008

Discussing/Age or gender category:	Smoking	Alcohol	Nutrition	Physical activity*	Total
0-19 years	30 (3.6%)	4 (0.5%)	108 (13%)	131 (15.8%)	831

20-44 years	165 (6.3%)	66 (2.5%)	237 (9%)	415 (15.8%)	2633

45-64 years	148 (8.6%)	65 (3.8%)	203 (11.8%)	308 (17.9%)	1722

65-74 years	40 (6.4%)	19 (3.1%)	74 (11.9%)	112 (18%)	622

75-84 years	15 (4.7%)	6 (1.9%)	42 (13%)	65 (20.2%)	322

>85 years	2 (2.7%)	1 (1.4%)	5 (6.8%)	15 (20.3%)	74

Male (ref)	197 (8.2%)	102 (4.3%)	242 (10.1%)	466(19.5%)	2389

Female	203 (5.3%)**	79 (2.1%)**	426 (11.2%)	579 (15.2%)**	3808

*Significant trend: discussing of lifestyle behaviour increases when patient is older, logistic regression, OR: 1.01 & CI 1.01-1.01. **Significant difference, logistic regression (male as reference group), Smoking = OR: 0.6 & CI: 0.5-0.8; Alcohol = OR: 0.4 & CI: 0.3-0.5; Physical activity = OR: 0.7 & CI: 0.6-0.8.

GPs discuss smoking, alcohol and nutrition with patients from different educational backgrounds equally. GPs discuss physical activity significantly more with patients with a college or university degree compared to patients with no formal education.

GPs discuss smoking and alcohol behaviour most with patients between 45 and 64 years of age. Nutrition is discussed equally with patients in all age categories.

The likelihood that GPs discuss physical activity increases with older patients (Odds ratio 1.01, 95% CI 1.01-1.01).

GPs discuss smoking, alcohol and physical activity significantly more with male than with female patients. Nutrition is discussed almost equally with male and female patients.

## Discussion

This study is, to the best of our knowledge, the first to compare the frequency of discussing lifestyle behaviour during primary care consultations from 1975 until 2008. Our results demonstrate that smoking behaviour and physical activity were discussed somewhat more often during consultations in more recent years (especially since 1989). Whether nutrition and alcohol use are more often discussed over the years can not be confirmed.

It is possible that the increased policy attention for a healthy lifestyle of recent years has led to more awareness of and discussion about lifestyle habits in primary care consultations. Conceivably, the introduction of the Public Health Collective Prevention Act WCPV in 1989 in the Netherlands also focussed greater attention on a healthy lifestyle. This legislation supports the protection and promotion of public health for specific groups and it also promotes the prevention and early detection of diseases. In 2008, this law was replaced by the Public Health Act [33].

This study shows that overall physical activity was the most discussed and alcohol use the least discussed during primary care consultations. This is consistent with a previous Swedish study [14]. Besides, other research indicates that advice from GPs on alcohol behaviour is less common than advice about smoking, nutrition or exercise [34].

Although our results show that most lifestyle behaviours were discussed (somewhat) more in more recent years they still feature in only a minority of consultations. Theoretically, of course, it is possible that lifestyle behaviour had been discussed in a previous consultation to the one recorded, or the GP may have planned to broach it in a later consultation.

Our study also suggests that, although the initiative to discuss lifestyle behaviour is only taken by GPs and patients in a small proportion of the consultations, both take the initiative to discuss lifestyle behaviour. In the case of smoking cessation and alcohol use GPs are more likely to broach the subject, while patients bring up their physical activity behaviour more often during consultations.

In addition, our results show that GPs discuss lifestyle behaviour about smoking, alcohol and nutrition with patients from different educational backgrounds equally. GPs discuss physical activity even more with patients with a college or university degree compared to patients with no formal education. Apparently, the fact that unhealthy lifestyle has a higher prevalence in lower social economic groups does not result in more discussion about lifestyle behaviour with patients with a lower educational background during primary care consultations.

Furthermore, this study indicates that smoking behaviour, alcohol use and physical activity are more discussed with older, male patients. Nutrition, on the other hand, is discussed with almost as many male as female patients, from all age groups.

Additionally, our study demonstrates that GPs' approach to lifestyle behaviour did not change over time. Overall, it seems that GPs mostly use a 'symptom approach' to lifestyle advice; they discuss lifestyle behaviour when it is relevant for the patient's condition. For example, GPs discuss nutrition when the patient has a stomach ache and smoking cessation when the patient has breathing problems. Despite indications from previous research [14,15], GPs in our research did not focus on a 'high risk approach'. They discussed lifestyle behaviour with patients who were at risk or had a chronic disease, but this occurred not very often. Although it is possible that GPs who started with a 'symptom' to discuss lifestyle behaviour may have chosen to do this in those patients who are also at high risk.

A population approach to lifestyle advice is not visible in our consultations, since GPs discuss lifestyle behaviour with only a minority of their patients. This is in line with a previous study by Lawlor et al. [16], which also found that GPs do not take a population approach and are therefore unlikely to affect population health.

In the Netherlands, the UK and other western countries it is common that GPs delegate tasks, regarding patients with chronic diseases and their lifestyle, to practice nurses, nurse physicians or assistants [35,36]. Although this form of task delegation is important to help chronically ill patients, these professionals generally do not provide prevention advice to patients who do not yet have risk symptoms. An opportunity to tackle this problem lies in the introduction of prevention consultations in the Netherlands, performed by GPs, nurse physicians or practice nurses. The goal of the prevention consultation is to detect patients early who are at risk for conditions such as heart and vascular disease, diabetes or kidney disease. These are patients who are not (yet) at risk but during prevention consultations, they have the opportunity to discuss their lifestyle behaviour. The precise design of the prevention consultation is still under construction, but it will most likely be introduced during 2011 [17,37].

## Strengths and limitations

A major strength of the present study is that we had access to data from video-recorded consultations between 1975 and 2008. Furthermore, observations are a more reliable source than self-reporting by GPs or patients, which could be biased. Besides, neither patients nor GPs were aware of the fact that the analysis would focus on communication about lifestyle behaviour.

Some limitations should also be noted. First, we did not examine the content of lifestyle behaviour during consultations. Therefore it was not possible to say anything about the quality of the discussion of lifestyle behaviour between GPs and patients. However, our data contains both simple (routine) questions about lifestyle behaviour (like 'do you smoke?') and extensive advice about lifestyle behaviour. Future research should investigate the content and quality of discussing lifestyle behaviour.

Second, observers who coded the discussion of 'physical activity' did not code physical activity primarily as a lifestyle topic. As a result, the frequency of discussion on physical activity also includes references to posture, exercise and sports in general. This could explain a higher frequency of discussion on physical activity for all years.

Furthermore, more female GPs participated in the later studies. We did not correct for this variable, since it is in line with the rising number of female GPs in the Netherlands in recent years [38]. As we do not know of any research indicating gender difference in discussing lifestyle factors, we do not expect that this increase of female GPs in more recent years has had any impact on our findings.

Although patients' response for the different studies was high (between 77% and 88%) and several studies show no difference between responders and non-responders regarding age and gender [26,28,29], there are differences between responders and non-responders in other studies. In one study non-responders were somewhat older [30] and in two studies more female patients did not consent [24,30]. In some studies patients who did not consent showed also more psychological or social complaints [24,26] or female genital symptoms [28] than those who did consent.

Moreover, we were not aware of the present lifestyle behaviour of the patient, except when it was discussed during a consultation. Therefore, we could not exclusively select patients who were at high risk and in need of lifestyle advice. Furthermore, we did not identify consultations in which it was not appropriate to discuss lifestyle behaviour (such as palliative care or breaking bad news consultations). Further research could elaborate these issues.

In addition, an important limitation of our cross sectional design is the inability to attribute cause and effect. For example we state that unhealthy lifestyles are more prevalent in low SES groups but this does not result in more elaborate discussion of these issues. It is also possible that they are more prevalent because they are not discussed by the GP.

Lastly, previous research shows that the number of people with obesity is increasing over time [7,39], which could automatically lead to greater attention for physical activity during consultations, possibly weakening our findings about the increase in discussing physical activity over time. On the other hand, we may have underestimated the effect of discussing smoking behaviour with patients during consultations since the number of smokers has declined in recent years [40] and our results show an increase in discussing smoking behaviour.

## Conclusion

In recent years there is greater awareness of a healthy lifestyle, which is reflected to a small extent in the higher frequencies of discussing smoking and physical activity behaviour over time. It is less clear whether or not nutrition and alcohol use are also more often discussed in recent years. Moreover, lifestyle choices (especially about alcohol use) are still discussed in only a minority of consultations. GPs mainly discuss lifestyle behaviour when relevant to the patient's condition and do not discuss lifestyle behaviour as a routine procedure i.e. do not use it for primary prevention. Moreover, our study showed that GPs' approach to lifestyle behaviour did not change over time. These findings highlight the importance of introducing prevention consultations, which will enable people who do not (yet) have risk symptoms to discuss their lifestyle behaviour.

## Competing interests

The authors declare that they have no competing interests.

## Authors' contributions

SvD conceived and supervised the study and helped to draft the manuscript. PV co-ordinated the study and helped to draft the manuscript. JN carried out the study and drafted the manuscript. All authors read and approved the final manuscript.

## Pre-publication history

The pre-publication history for this paper can be accessed here:

http://www.biomedcentral.com/1471-2296/11/87/prepub
